# Inflammatory laboratory findings associated with severe illness among hospitalized individuals with COVID-19 in Medan, Indonesia: a cross-sectional study

**DOI:** 10.12688/f1000research.74758.1

**Published:** 2021-12-06

**Authors:** Darmadi Darmadi, Cennikon Pakpahan, Riska Habriel Ruslie, Andri Rezano

**Affiliations:** 1 Department of Internal Medicine, Faculty of Medicine, Universitas Sumatera Utara, Medan, North Sumatera, Indonesia; 2 Andrology Study Program, Department of Biomedical Sciences, Faculty of Medicine, Universitas Airlangga, Surabaya, East Java, Indonesia; 3 Department of Child Health, Faculty of Medicine, Universitas Prima Indonesia, Medan, North Sumatera, Indonesia; 4 Department of Biomedical Sciences, Faculty of Medicine, Universitas Padjadjaran, Sumedang, West Java, Indonesia

**Keywords:** COVID-19, inflammatory, cytokine, comorbid, good health, well-being, vaccination

## Abstract

**Background: **Coronavirus disease (COVID-19) is still a global health problem. COVID-19 patients with severe pneumonia have a higher risk for critical illness, mostly complicated by acute respiratory distress syndrome. The inflammatory response is critical, and the cytokine storm increases severity of COVID-19. Many factors could be associated with a cytokine storm but these are incompletely understood.

The aim of this study is to present characteristics of patients with COVID-19 and explore the clinical and inflammatory parameters of severe and critically ill COVID-19 patients in the intensive care unit (ICU).

**Method: **The cross-sectional study was conducted in all severe COVID-19 patients admitted to the ICU. Peripheral blood was taken for laboratory examination within 24 hours of admission. Hematologic parameters, serum electrolyte, renal function, liver function, pancreas enzyme, D-dimer, inflammatory cytokines interferon (IFN)-gamma, tumor necrosis factor (TNF)-alpha, interleukin (IL)-6, IL-10, monocyte chemoattractant protein-1 (MCP-1), and C-reactive protein (CRP) were assessed in this study. Comparison analyses were done between sex, comorbidity existed, body mass index (BMI), and vaccination status.

**Results: **A total of 80 subjects were included in the study. The most frequent comorbidities found among the subjects were obesity (36.35%) and diabetes (22.5%). Only 13.75% of subjects were vaccinated. Laboratory results indicated leukocytosis and neutrophilia, with neutrophil-lymphocyte-ratio (NLR) of 7. The mean inflammatory findings (IL-6, IL-10, TNF-alpha, IFN-gamma, MCP-1), D-dimer, CRP, and lipase increased. Lipase levels were higher in men (p=0.003) and in comorbidity groups. No significant differences found with different BMI groups. Lipase, IL-6, and MCP-1 levels were significantly higher (p=0.019, <0.0001, and 0.03, respectively) in the non-vaccinated group.

**Conclusions: **Most patients with severe COVID-19 have comorbidities and increased inflammatory markers.

## Introduction

Severe acute respiratory syndrome coronavirus 2 (SARS-CoV-2) was the cause of the catastrophic coronavirus disease (COVID-19) pandemic that began in January 2020. ^1^^,^^2^ It has claimed 4.4 million human lives as of 22 August 2021. ^3^ By August 2021, 4,043,736 cases were reported in Indonesia with 130,182 deaths. ^4^ COVID-19 has a fatality rate of 2.3%, less than both the severe acute respiratory syndrome coronavirus outbreak (SARS-CoV) (9.5%) in 2003 and the Middle East respiratory syndrome coronavirus outbreak (MERS-CoV) (34.4%) in 2012. ^5^ The outbreak initially was linked to the Hua Nan seafood and wet animal market in Wuhan. ^6^

The SARS-CoV-2 infection varies from asymptomatic, mild upper respiratory tract illness, and severe pneumonia with respiratory failure and death. ^7^ Severe patients with COVID-19 usually present with respiratory rates greater than 30 breaths/minutes, oxygen saturation (SpO _2_) less than 93%, and greater than 50% lung infiltrates, and are at higher risk for clinical deterioration and critical illness. ^8^ Acute respiratory distress syndrome (ARDS) was the most common complication occurring in 60-70% of patients admitted to the intensive care unit (ICU). ^9^ ARDS occurs most often in the setting of pneumonia, sepsis, aspiration of gastric contents or severe trauma and is present in ~10% of all patients in intensive care units worldwide. ^10^ This wide range of differences is presumably caused by the atypical disease process in ARDS, suggesting non-effectivity of mechanical ventilation in reducing lung injury. ^11^ Mortality outcomes could be influenced by age, sex, race, chronic illness, comorbidities, insurance, geographic location, and medical management. ^12^^–^^15^

The inflammatory response plays a critical role in COVID-19, and the inflammatory cytokine storm increases the severity of COVID-19. ^16^^,^^17^ Periphery blood inflammatory factors such as interferon (IFN)-gamma, tumor necrosis factor (TNF), interleukin (IL)-10, IL-6, and monocyte chemoattractant protein-1 (MCP-1) may increase during COVID-19 infection. ^18^^–^^20^ Many factors including sex, body mass index (BMI), comorbidity, and vaccination status could be associated with the incidence of the cytokine storm and severe COVID-19. ^18^^,^^21^^–^^23^ The cytokine storm is crucial to the progression of COVID-19 and might lead to ARDS and death. ^24^ Patients who survive from cytokine storms tend to suffer long-term lung damage and fibrosis, causing impairment in pulmonary function and lower quality of life. ^25^

In Indonesia, resources for the management of COVID-19, particularly laboratory parameters are still constrained. This issue brought up the need for simple approaches to detect cytokine storms in patients with COVID-19, which could help stratify the risk of morbidity and mortality in COVID-19 patients at the time of hospitalization. In this study, we present details of patients with COVID-19 hospitalized in the ICU of Mitra Medica General Hospital in Medan, North Sumatera, Indonesia. We aim to explore the clinical and inflammatory parameters of severe and critically ill COVID-19 patients in the ICU.

## Methods

### Ethical approval

This study was approved by the ethics committee of Universitas Sumatera Utara (Ethical clearance number 453/KEP/USU/2020). The ethics committee is in charge for North Sumatera province including this study location. Informed consent was obtained before data collection. In this study, written informed consent was obtained from each patient’s proxy if the patient was unconscious. Otherwise, written informed consent was obtained from corresponding patient.

### Study and patients

The cross-sectional study was conducted in all COVID-19 cases (confirmed by the RT-PCR test) admitted to the ICU of Mitra Medica General Hospital Medan, Indonesia, between May and June 2021. Inclusion criteria were all subjects classified as severe COVID-19 according to the World Health Organization guidelines. ^26^ Diagnosis of severe COVID-19 was made if subjects met one or more of the following criteria: dyspnea, respiratory rate of 30/min, SpO _2_ of 93%, PaO _2_/FiO _2_ ratio less than 300 mmHg, greater than 50% lung infiltrate on CT scan within 24-48 hours, and those with respiratory failure, septic shock, and/or multiple organ dysfunction. ^26^

### Data collection

Demographic data, clinical history, and vaccination status of patients were collected from the patient’s medical record. BMI data were calculated from the patient’s weight and height. A total of 10 mL peripheral blood was taken for laboratory examination within 24 hours after the patient was admitted to the ICU. Laboratory parameters included in this study were hematologic parameters (hemoglobin, leukocyte, thrombocyte, neutrophil, lymphocyte, monocyte), serum electrolyte (sodium, potassium, chloride, calcium), renal function (urea, creatinine), liver function [aspartate transaminase (AST); alanine transaminase (ALT)], pancreas enzymes (amylase, lipase), D-dimer, inflammatory cytokines (IFN-gamma, TNF-alpha, IL-6, IL-10, MCP-1), and C-reactive protein (CRP).

The inflammatory cytokines were analyzed with the following kits: IL-6, Human IL-6 Quantikine ELISA kit Immunoassay (R&D System, Minneapolis, MN, USA); IL-10, Human IL-10 Quantikine ELISA kit Immunoassay (R&D System, Minneapolis, MN, USA); MCP-1, Human CCL2/MCP-1 Quantikine ELISA kit Immunoassay (R&D System, Minneapolis, MN, USA); IFN-gamma, Human IFN-gamma Quantikine ELISA kit Immunoassay (R&D System, Minneapolis, MN, USA); TNF-alpha, Human TNF-alpha Quantikine ELISA kit Immunoassay (R&D System, Minneapolis, MN, USA).

### Statistical analysis

Statistical analysis was done using GraphPad Prism version 8.0. Normality test with Kolmogorov-Smirnov test was conducted to determine distribution normality of the data. Parametric data were presented in mean ± standard deviation, while non-parametric data were presented as median and interquartile range. Data were compared between genders, subjects with comorbidity and without comorbidity, BMI, and vaccination status. Patients’ BMI were classified as underweight, normal weight, overweight, and obese based on BMI criteria for Asia. ^27^ The differences between two groups were tested with the independent t-test and the Mann-Whitney test. T-test was utilized for parametric data and Mann-Whitney test for non-parametric one. Meanwhile, differences between groups of more than two were done with the one-way ANOVA test for parametric data and otherwise with the Kruskal Wallis test. Statistical analysis was done within 95% confidence interval. Significance was established based on *p-*value of <0.05.

## Results

### Patient demographics and clinical features

A total of 80 subjects were included in the study. The demographics data is presented in Table 1. The mean age of all the subjects was 59 years old. There were more male subjects. The most frequent comorbidity found among the subjects was obesity (36.35%), followed by diabetes (22.5%). Only 11 subjects (13.75%) were vaccinated in this study.

**Table 1.** Demographic of patients infected with severe SARS-CoV-2.

Variable	Total (n = 80), n (%)
**Age, in years (mean ± standard deviation)**	59.93 **± 8.78**
**Gender**	
Male	48 (60)
Female	32 (40)
**Comorbidity**	
Diabetes	18 (22.5)
Obesity	29 (36.35)
Cardiovascular comorbid	4 (5)
Hypertension	13 (16.25)
Stroke	4 (5)
Chronic kidney disease	4 (5)
Pulmonary Disease (chronic obstructive pulmonary disease, tuberculosis)	4 (6.25)
**Vaccination status**	
Vaccinated	11 (13.75)
Non-vaccinated	69 (86.25)

### Laboratory findings

**Figure 1.**
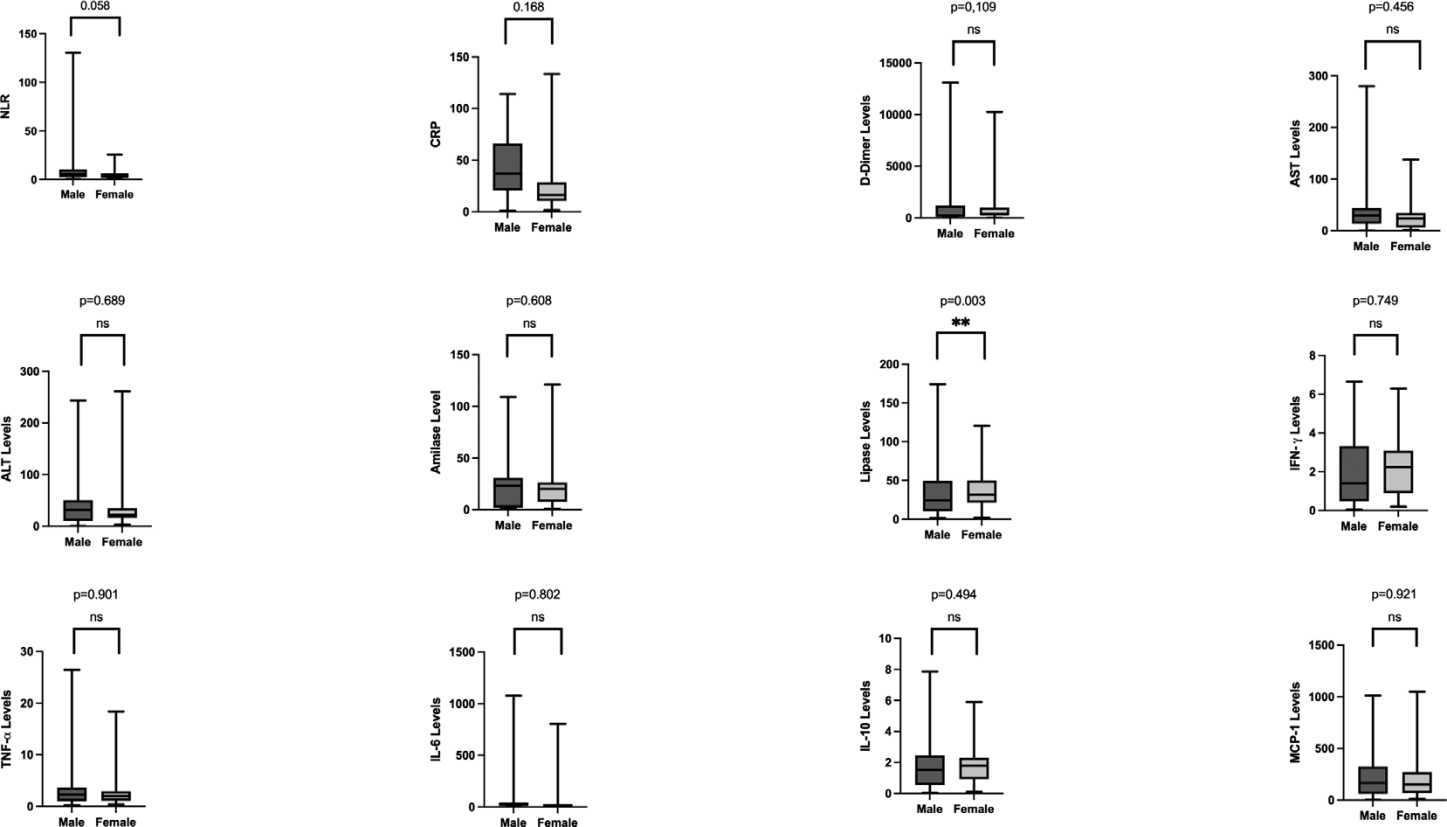
Laboratory parameters between gender. Lipase levels were significantly higher in men (129.5 (±52.32), p = 0.003). C-reactive protein, Aspartate aminotransferase levels, and interferon-gamma were also higher in men. Women, though not significant, had higher levels of D-dimer, Alanine Aminotransferase levels, amylase, tumor necrosis factor-alpha, interleukin-6, interleukin-10, and monocyte chemoattractant protein-1.

**Figure 2.**
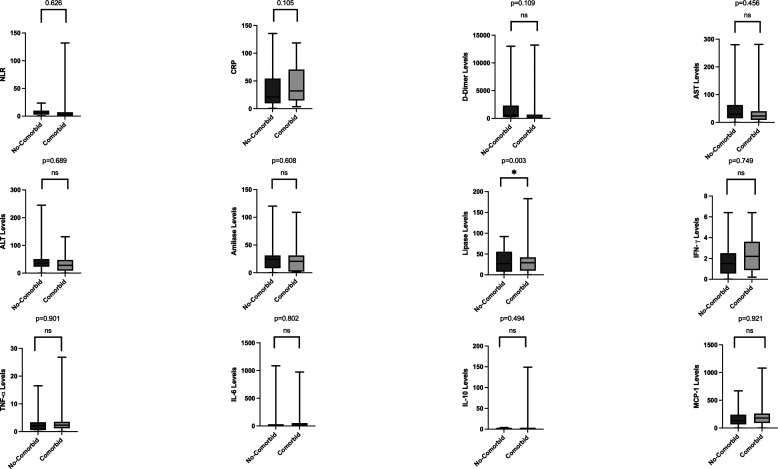
Laboratory parameters between non-comorbid and comorbid groups. Lipase levels were significantly higher in patients with comorbidity. Most laboratory parameters were higher in patients with comorbidities, except for aspartate aminotransferase levels and Interferon-gamma.

**Figure 3.**
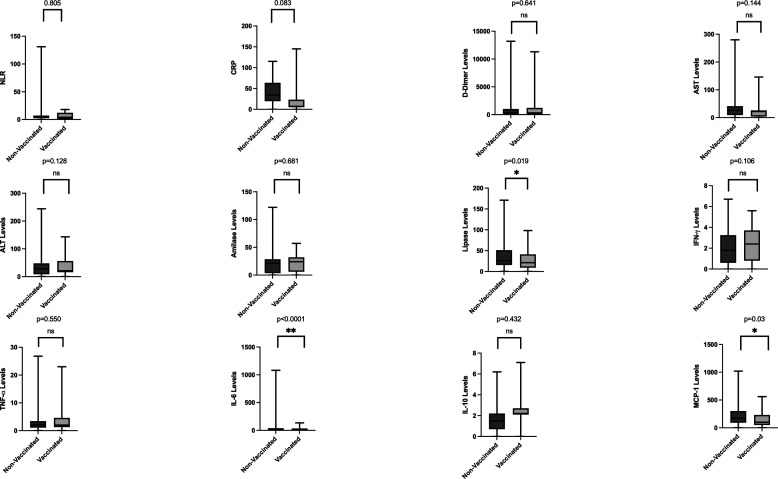
Laboratory parameters between non-vaccinated and vaccinated groups. Lipase levels, interleukin-6 levels, and monocyte chemoattractant protein-1 levels were significantly lower in vaccinated patients ( *p-*value = 0.019, <0.0001, and 0.03, respectively). Aspartate Aminotransferase levels and amylase levels also were lower in vaccinated subjects.

Table 2 presents the laboratory results from this study. Leucocyte and neutrophil percentage increased in the subjects. Neutrophil to lymphocyte ratio (NLR) was 7. The inflammatory findings were increased in severe COVID-19 patients in the study compared to normal value. D-dimer as a coagulopathy parameter increased above the normal range in this study. CRP, ALT levels, AST levels, and lipase also increased in the subjects. Other parameters including serum electrolyte levels and renal function were relatively within normal reference value. Results of comparison analysis between males and females are shown in Figure 1. Lipase levels were higher in men (129.5 (±52.32), p = 0.003). Analyses between BMI groups are presented in Table 3. There are no significant differences found between different BMI groups. As for analysis regarding non-comorbid and comorbid groups, lipase levels were higher in groups with comorbidity compared to those without comorbidity (shown in Figure 2). Between the vaccinated and non-vaccinated groups, results indicated a significantly higher level of lipase, IL-6, and MCP-1 ( *p-*value = 0.019, <0.0001, and 0.03, respectively) in the non-vaccinated group ( Figure 3).

**Table 2.** Clinical and laboratory findings of severe SARS-CoV-2.

Variables	Baseline	Normal value
BMI (Body Mass Index) (kg/m ^2^)	23.60 (23.40-24.60)	18.5-23
Hemoglobin (g/dL)	12.25 (11.80-13.00)	12.5-16.3
Leukocyte (per mm ^3^)	11,300 (9,990-12,300)	4,000-10,200
Thrombocyte (per mm ^3^)	294,000 (257,000-332,000)	150,000-450,000
Neutrophil (%)	80.70 (78.70-84.60)	55-70
Lymphocyte (%)	12.20 (8.5-15.1)	20-40
Monocyte (%)	4.6 (4.2-5.8)	2-8
C-reactive protein (mg/L)	77 (64-96)	<10
Sodium/Na (mmol/L)	138.5 (135-140)	135-145
Potassium/K (mmol/L)	4.5 (2.8-7.2)	3.5-5
Chloride (mEq/L)	105.5 (91-119)	95-105
Calcium (mmol/L	8.9 (8.8-9.2)	8.6-10.3
Urea (mg/dL)	44 (33-54)	15-40
Creatinine (mg/dL)	1.12 (0.94-1.35)	0.7-1.2
D-Dimer (ng/mL)	800 (760-980)	<500
Aspartate Aminotsransferase (U/L)	55.5 (44-65)	<31
Alanine Aminotransferase (U/L)	67 (46-73)	<32
Amylase (U/L)	57 (50-58)	19-86
Lipase (U/L)	113.5 (106-135)	7-59
Interferon-Gamma (pg/mL)	4.4 (4-5)	<4.2
Tumor necrosis factor-alpha (pg/mL)	7.3 (6.2-8.4)	<2.8
Interleukin-6 (pg/mL)	43.5 (32-57)	<7.0
Interleukin-10 (pg/mL)	5.8 (5.2-6.8)	<3.5
Monocyte chemoattractant protein-1 (pg/mL)	380 (295-455)	<300

All data presented as median (interquartile range).

**Table 3.** Inflammation laboratory findings of hospitalized patients infected with SARS- Cov-2 stratified by body mass index.

Parameter	Underweight	Norm weight	Overweight	Obese	p-value
Neutrophil-lymphocyte ratio	3.5 (1.5-10.75)	7.0 (3.0-18.0)	9.5 (4.5-15.0)	6.0 (2.0-15.5)	0.472
C-reactive protein	67.0 (59.25-133.3)	70.0 (55.0-148.0)	92.5 (64.25-151.0)	58.0 (49.5-147.0)	0.632
D-dimer	2180 (377.5-11.400)	900 (560-1800)	840 (540-2750)	800 (500-1475)	0.939
Alanine Aminotransferase	44.5 (30.75-264.5)	55 (34-79)	70 (27.3-129.0)	54 (30.5-78.0)	0.656
Aspartate Aminotransferase	34 (19.50-86.0)	67 (36-88)	62.5 (32.5-127.3)	69.0 (33.5-98.0)	0.697
Amylase	66 (42.0-88.5)	55 (34-66)	67.0 (55.2-84.7)	53.0 (33.5-98.0)	0.072
Lipase	154 (120.5-177.0)	115 (106-186)	109.5 (70.5-140.8)	106.0 (82.0-138.0)	0.246
Interferon-gamma	3.7 (2.17-9.05)	4.9 (3.2-7.8)	4.35 (3.02-5.8)	4.3 (2.35-7.10)	0.667
Tumor necrosis factor-Alpha	5.6 (5.07-19.33)	7.9 (6.0-11.9)	7.35 (5.17-10.08)	7.3 (5.05-10.0)	0.676
Interleukin-6	26.0 (20.7-854.0)	49.0 (26.0-78.0)	44.5 (25.7-71.5)	43.0 (21.5-100)	0.964
Interleukin-10	5.3 (4.5-5.4)	6.2 (4.6-7.9)	5.5 (4.0-7.75)	6 (4.3-8.6)	0.632
Monocyte chemoattractant protein-1	275.0 (222.5-650)	315 (185-505)	460 (352.5-602.5)	355.0 (185.0-580)	0.232

All data presented as median (interquartile range).

## Discussion

More men were included in our study group with severe COVID-19, suggesting that they suffer from the severe form of COVID-19 compared with women. Most recent studies suggested that men also tend to present more severe forms of the disease and have a higher rate of mortality. ^28^ The number of men is 2.4 times that of women in the deceased patients. While men and women had the same susceptibility, men were more prone to dying. ^28^^,^^29^ Potential risk factors have been suggested as different behavior between genders, genetic and hormonal factors, and the influence of sex genetics in viral pathogenesis. ^29^ Risky behaviors such as smoking and alcohol consumption have been reported in more men than women, ^30^ and these behaviors increase risks for hypertension, cardiovascular disease, and chronic pulmonary disease, which could exacerbate severity and susceptibility to COVID-19. The mechanism of SARS-CoV-2 infection is regulated by the expression of ACE-2 and TMPRSS2 genes. These factors are often associated with sex, for instance the ACE-2 gene is found on the X-chromosome. Inactivation of this gene has been associated with the incidence of COVID-19 in males and females. ^31^ The gene that transcribes TMPRSS2 is influenced by androgens that the presence of androgens promote the expression of TMPRSS2. TMPRSS2 act as co-receptor for SARS-CoV-2 cell invasion and it’s high expression will lead to increased susceptibility for COVID-19. ^32^^,^^33^

The mean age of all the subjects in our study was 59 years old, with the youngest subject over 50 years old. Older people were more sensitive to SARS-CoV-2 infection and had a higher positive rate than younger age. ^34^ Older subjects are also associated with increased mortality from COVID-19 due to poorer lung function and the likelihood of comorbidities when compared to younger patients. ^34^^,^^35^ Aging significantly causes an increase in pro-inflammatory cytokine levels (CRP, D-dimer, Procalcitonin, and IL-10). ^36^ Older patients normally experience a decline in physiological immune function and immunosuppression, thus making it difficult for them to control pro-inflammatory responses. ^35^

Obesity is one comorbidity related to severe COVID-19. ^37^ Subjects with a BMI of less than 18.5 kg/m ^2^ and greater than 25 kg/m ^2^ have a higher risk for fatal illness. ^38^ In this study, a total of 36.35% were obese and 6.25% were underweight based on Asian BMI criteria. Obesity is one of the risk factors for cardiometabolic disease and is reported to cause immune system dysregulation. Obese patients have the highest risk for longer hospitalization and death due to COVID-19. ^39^ Adipocytes could increase the inflammatory response by stimulating macrophages to produce interleukins (IL-1, IL-6, IL-8, IL-10) and TNF-alpha. Meanwhile, underweight COVID-19 patients are at risk of developing acute kidney injury, which could worsen the patient’s condition. ^40^ In this study, laboratory parameters were not significantly different within various BMI groups. This might also be influenced by other conditions of the patients.

Diabetes mellitus is also widely associated with the incidence of COVID-19. ^41^ Increased glucose metabolism in patients with diabetes could directly enhance the replication of the SARS-CoV-2. Increased glucose escalates the production of mitochondrial reactive oxygen species and activates hypoxia-inducible factor 1α. ^42^ Insulin resistance itself is associated with impaired response to IFN type 1, thus generating a high viral load and inhibiting body inflammatory response. ^43^

Vaccination is part of the prevention program against SARS-CoV-2. The vaccine promotes antibody production to prevent COVID-19. ^44^ Administration of the vaccine also effectively reduces the severity of the disease. ^23^ In this study, 86.25% of patients were reported as non-vaccinated. Several parameters were significantly different between the vaccinated and non-vaccinated groups. Lipase, IL-6, and MCP-1 were higher in the non-vaccinated group. Increased lipase and IL-6 indicate an inflammatory response and imply more severe disease. ^7^ On the other hand, MCP-1 is suspected to be related to inhibition of IFN-signalling. ^45^ IFNα and IFN-β have antiviral activity; thus non-vaccinated individuals tend to have poorer immune response due to low antiviral activity. ^46^

Subjects in this study had an increased leukocyte count and lymphopenia. This finding was also found in the meta-analysis by Huang *et al*., which reported patients with severe COVID-19 tended to have higher leukocyte counts and lower lymphocyte counts compared to non-severe illness. ^47^ Leucocytosis may be present due to co-infection with bacterial pneumonia, and steroid medication given to those with severe illness is known to induce leucocytosis or variability in the immune response. ^48^ Lymphopenia decline might be directly induced by lymphocyte tissue destruction, inflammatory cytokines or metabolic disorder that caused by COVID-19 infection. TNF-alpha, IL-6, and other inflammatory cytokines could induce deficiency in lymphocytes. ^49^

Neutrophilia was also reported in this study, with a NLR of 7. In patients with COVID-19, NLR may reflect the severity of inflammation. Neutrophil percentages have been seen to be mostly within normal range in non-severe cases but were increased in severe form of illness. ^48^ Elderly and critical patients tend to present with neutrophilia, and this condition is suggested to be related to the cytokine storm. ^50^^,^^51^ A risk predictive model by Liu *et al*. suggested that incidence of severe disease was 50% in patients with an age 50 years or older and NLR greater or equal to 3.13 compared to 9.1% in patients with an age 50 years or older and NLR less than 3.13. ^52^ In our study, subjects were >50 years and NLR >3.13, thus correlating with the risk of severe disease.

Inflammatory parameters (IL-6, IL-10, TNF-alpha, and IFN-gamma, MCP-1) were increased in COVID-19 patients with severe illness in this study. These findings were also found in recent studies. ^16^^,^^20^^,^^24^^,^^53^^,^^54^ Patients with COVID-19 had high amounts of pro-inflammatory cytokines (IFN-gamma, TNF-alpha, IP-10, IL-1B, MCP-1). Patients requiring ICU admission had even higher number of cytokines, suggesting a cytokine storm was associated with progression of ARDS and severe illness. However, COVID-19 patients also present with increased anti-inflammatory cytokines (IL-4, IL-10), which differs from SARS-CoV infection. ^24^^,^^53^ The Univariate Cox Analysis by Yang *et al*. indicated that circulating IL-6 significantly predicted the progression of COVID-19 infection. Serum IL-6 was higher in COVID-19 patients with pneumonia compared to those without pneumonia. Increased IL-6 might induce tissue-damaging-inflammation and cause alveolar cell injury. ^20^ Patients with IL-6 greater than 32.1 pg/mL were more likely to have severe complications. ^16^ IL-6 trans-signaling could enhance production of IL-8, MCP-1, and IL-10. ^54^

D-dimer also increased in our subjects, with a mean of 800 ng/mL. D-dimer is a fibrin degradation product widely used as a biomarker for thrombotic disorders. A D-dimer value less than 500 ng/mL is usually considered normal. D-dimer can predict severe and fatal cases of COVID-19 with moderate accuracy (sensitivity 77%, specificity 71%). ^55^ In a multicentre meta-analysis by Paliogiannis *et al*., D-dimer concentrations in patients with severe COVID-19 are significantly higher when compared to those with non-severe forms. ^56^ In the analysis by Ozen *et al*., threshold D-dimer value of 370 ng/ml was calculated to have 74% specificity and 77% sensitivity for predicting lung involvement in patients with COVID-19. ^57^ A cut-off of 1,500 ng/mL is the optimal value of admission D-dimer for predicting mortality in COVID-19 patients. ^58^

CRP levels increased almost eightfold above reference values in this study. CRP is an active regulator of host innate immunity and induces the classical complement pathway, and is thereby capable of mediating inflammation. ^59^ A significant increase of CRP was found in COVID-19 patients, with levels on average of 20 to 50 mg/L. ^60^ CRP is normally not elevated in viral infections, but the macrophage activation syndrome may explain the high serum CRP and poorer disease progression. Elevated CRP may also indicate co-infections of bacterial etiology. ^61^ Up to 86% of patients with a severe form of COVID-19 had increased CRP, in higher concentration compared to mild or non-severe patients. ^62^ The risk of developing severe events is increased by 5% for every one-unit increase in CRP levels in COVID-19 patients. ^63^

The mean level of lipase was significantly higher in men in our study. This finding was similar to the study by Barlass *et al*, who showed that increased lipase indicated possible inflammation of the pancreas and was connected with poor prognosis. ^64^ Although there was higher lipase activity in the male animal model there was no definite explanation for higher activity of lipase in men. ^65^ Lipase levels were also significantly different in the group of patients with and without comorbidities. After adjusted analysis with groups of comorbidities, there was no significant difference. Perhaps comorbidities could interfere with the body’s physiological processes and induce stress in various organs, including the pancreas.

This study has limitations because we don’t compare the laboratory profiles between severe groups and mild/moderate groups. However, for patients with COVID-19, the presence of comorbidities and elevated inflammatory markers should raise healthcare provider’s awareness for the risk of severe disease course. There are also interesting results that could be important for future treatment. Lipase, IL-6, and MCP-1 results were found significantly different between the vaccine and non-vaccine groups. Elevated lipase may indicate possible pancreatic involvement that may be a consideration in the management of COVID-19.

## Data availability

### Underlying data

Figshare: COVID Master Data ICU.xlsx, https://doi.org/10.6084/m9.figshare.16823611.v2. ^66^

Data are available under the terms of the Creative Commons Zero “No rights reserved” data waiver (CC0 1.0 Public domain dedication).
