# Inflammatory laboratory findings associated with severe illness among hospitalized individuals with COVID-19 in Medan, Indonesia: a cross-sectional study

**DOI:** 10.12688/f1000research.74758.2

**Published:** 2022-01-14

**Authors:** Darmadi Darmadi, Cennikon Pakpahan, Riska Habriel Ruslie, Andri Rezano

**Affiliations:** 1 Department of Internal Medicine, Faculty of Medicine, Universitas Sumatera Utara, Medan, North Sumatera, Indonesia; 2 Andrology Study Program, Department of Biomedical Sciences, Faculty of Medicine, Universitas Airlangga, Surabaya, East Java, Indonesia; 3 Department of Child Health, Faculty of Medicine, Universitas Prima Indonesia, Medan, North Sumatera, Indonesia; 4 Department of Biomedical Sciences, Faculty of Medicine, Universitas Padjadjaran, Sumedang, West Java, Indonesia

**Keywords:** COVID-19, inflammatory, cytokine, comorbid, good health, well-being, vaccination

## Abstract

**Background: **Coronavirus disease (COVID-19) remains a global health problem. COVID-19 patients with severe pneumonia have a higher risk for critical illness, mostly complicated by acute respiratory distress syndrome. The inflammatory response is critical, and the cytokine storm increases the severity of COVID-19. Many factors could be associated with a cytokine storm but they are incompletely understood.

This study presents characteristics of COVID-19 patients and explore the clinical and inflammatory parameters of severe and critically ill COVID-19 patients in the intensive care unit (ICU).

**Method: **This cross-sectional study was conducted in all severe COVID-19 patients admitted to the ICU. Peripheral blood was taken for laboratory examination within 24 hours of admission. Haematologic parameters, serum electrolyte, renal function, liver function, pancreas enzyme, D-dimer, inflammatory cytokines interferon (IFN)-gamma, tumour necrosis factor (TNF)-alpha, interleukin (IL)-6, IL-10, monocyte chemoattractant protein-1 (MCP-1), and C-reactive protein (CRP) were assessed in this study. Comparative analyses were done between sex, existing comorbidities, body mass index (BMI), and COVID-19 vaccination status.

**Results: **A total of 80 subjects were included in the study. The most frequent comorbidities found among the subjects were obesity (36.35%) and diabetes (22.5%). Only 13.75% of subjects were vaccinated. Laboratory results indicated leucocytosis and neutrophilia, with a neutrophil-lymphocyte-ratio (NLR) of 7. The mean inflammatory findings (IL-6, IL-10, TNF-alpha, IFN-gamma, MCP-1), D-dimer, CRP, and lipase increased. Lipase levels were higher in men (p = 0.003) and in comorbidity groups. No significant differences were found among different BMI groups. Lipase, IL-6, and MCP-1 levels were significantly higher (p=0.019, <0.0001, and 0.03, respectively) in the non-vaccinated group.

**Conclusions: **Most patients with severe COVID-19 have comorbidities and increased inflammatory markers.

## Introduction

Severe acute respiratory syndrome coronavirus 2 (SARS-CoV-2) was the cause of the catastrophic coronavirus disease (COVID-19) pandemic that began in January 2020. ^1^^,^^2^ It has claimed 4.4 million human lives as of August 22, 2021. ^3^ By August 2021, 4,043,736 cases were reported in Indonesia with 130,182 deaths. ^4^ COVID-19 has a fatality rate of 2.3%, less than the outbreaks of severe acute respiratory syndrome coronavirus outbreak (SARS-CoV) (9.5%) in 2003 and the Middle East respiratory syndrome coronavirus outbreak (MERS-CoV) (34.4%) in 2012. ^5^ The SARS-CoV-2 outbreak initially was linked to the Hua Nan seafood and wet animal market in Wuhan. ^6^

The SARS-CoV-2 infection varies from asymptomatic, mild upper respiratory tract illness, to severe pneumonia with respiratory failure and death. ^7^ Patients with severe COVID-19 usually present with respiratory rates greater than 30 breaths/minutes, oxygen saturation (SpO _2_) less than 93%, and greater than 50% lung infiltrates, and are at higher risk for clinical deterioration and critical illness. ^8^ Acute respiratory distress syndrome (ARDS) was the most common complication occurring in 60% to 70% of patients admitted to the intensive care unit (ICU). ^9^ ARDS occurs most often in the setting of pneumonia, sepsis, aspiration of gastric contents or severe trauma and is present in ~10% of all patients in ICU worldwide. ^10^ This wide range of differences is presumably due to the atypical disease process in ARDS, suggesting the non-effectivity of mechanical ventilation in reducing lung injury. ^11^ Mortality outcomes could be influenced by age, sex, race, chronic illness, comorbidities, insurance, geographic location, and medical management. ^12^^–^^15^

The inflammatory response plays a critical role in COVID-19. The inflammatory cytokine storm increases the severity of COVID-19. ^16^^,^^17^ Periphery blood inflammatory factors such as interferon (IFN)-gamma, tumour necrosis factor (TNF), interleukin (IL)-10, IL-6, and monocyte chemoattractant protein-1 (MCP-1) may increase during COVID-19 infection. ^18^^–^^20^ Many factors including sex, body mass index (BMI), comorbidities, and vaccination status could be associated with the incidence of the cytokine storm and severe COVID-19. ^18^^,^^21^^–^^23^ The cytokine storm is crucial to the progression of COVID-19 and might lead to ARDS and death. ^24^ Patients who survive from cytokine storms tend to suffer long-term lung damage and fibrosis, causing impairment in pulmonary function and lower quality of life. ^25^

In Indonesia, resources for the management of COVID-19, particularly laboratory parameters, remains constrained. This issue heightened the need for simple approaches to detect cytokine storms in patients with COVID-19, which could help stratify the risk of morbidity and mortality in COVID-19 patients at the time of hospitalisation. This study presents details of patients with COVID-19 hospitalised in the ICU of Mitra Medica General Hospital in Medan, North Sumatera, Indonesia. We aim to explore the clinical and inflammatory parameters of severe and critically ill COVID-19 patients in the ICU.

## Methods

### Ethical approval

This study was approved by the ethics committee of Universitas Sumatera Utara (Ethical clearance number 453/KEP/USU/2020). The ethics committee is in charge of the North Sumatera province including this study location. Informed consent was obtained before data collection. In this study, written informed consent was obtained from each patient’s proxy if the patient was unconscious. Otherwise, written informed consent was obtained from the corresponding patient.

### Study and patients

This cross-sectional study was conducted in all COVID-19 cases (confirmed by the RT-PCR test) admitted to the ICU of Mitra Medica General Hospital Medan, Indonesia, between May and June 2021. Inclusion criteria were all subjects classified as severe COVID-19 according to the World Health Organisation guidelines. ^26^ The diagnosis of severe COVID-19 was made if subjects met one or more of the following criteria: dyspnoea, respiratory rate of 30/min, SpO _2_ of 93%, PaO _2_/FiO _2_ ratio less than 300 mm Hg, greater than 50% lung infiltrate on CT scan within 24 to 48 hours, and those with respiratory failure, septic shock, and/or multiple organ dysfunction. ^26^

### Data collection

Demographic data, clinical history, and vaccination status of patients were collected from their medical records, including COVID-19 vaccination status. BMI data were calculated from the patient’s weight and height. A total of 10 mL peripheral blood was obtained for laboratory examination within 24 hours of admission to the ICU. Laboratory parameters included in this study were haematologic parameters (haemoglobin, leukocytes, thrombocytes, neutrophils, lymphocytes, monocytes), serum electrolyte (sodium, potassium, chloride, calcium), renal function (urea, creatinine), liver function [aspartate transaminase (AST); alanine transaminase (ALT)], pancreatic enzymes (amylase, lipase), D-dimer, inflammatory cytokines (IFN-gamma, TNF-alpha, IL-6, IL-10, MCP-1), and C-reactive protein (CRP).

The inflammatory cytokines were analysed with the following kits: IL-6, Human IL-6 Quantikine ELISA kit Immunoassay (R&D System, Minneapolis, MN, USA); IL-10, Human IL-10 Quantikine ELISA kit Immunoassay (R&D System, Minneapolis, MN, USA); MCP-1, Human CCL2/MCP-1 Quantikine ELISA kit Immunoassay (R&D System, Minneapolis, MN, USA); IFN-gamma, Human IFN-gamma Quantikine ELISA kit Immunoassay (R&D System, Minneapolis, MN, USA); TNF-alpha, Human TNF-alpha Quantikine ELISA kit Immunoassay (R&D System, Minneapolis, MN, USA).

### Statistical analysis

Statistical analysis was done using GraphPad Prism version 8.0. A normality test with the Kolmogorov-Smirnov test was conducted to determine the distribution normality of the data. Parametric data were presented in as means ± standard deviations, while non-parametric data were presented as medians and interquartile ranges. Data were compared between genders, subjects with comorbidities and without comorbidities, BMI, and vaccination status. Patients’ BMIs were classified as underweight, normal weight, overweight, and obese based on BMI criteria for Asia. ^27^ The differences between the two groups were tested with the independent t-test and the Mann-Whitney test. The t-test was utilised for parametric data and the Mann-Whitney test for non-parametric data. Meanwhile, differences between more than two groups were done with the one-way ANOVA test for parametric data and otherwise with the Kruskal Wallis test. Statistical analysis was performed within 95% confidence intervals. Significance was established based on *p-*values of <0.05.

## Results

### Patient demographics and clinical features

A total of 80 subjects were included in the study. The demographic data are presented in Table 1. The mean ages of all the subjects was 59 years old, and most were male. The most frequent comorbidity found among the subjects was obesity (36.35%), followed by diabetes (22.5%). Only 11 subjects (13.75%) were vaccinated in this study.

**Table 1.** Demographic of patients infected with severe SARS-CoV-2.

Variable	Total (n = 80), n (%)
**Age, in years (mean ± standard deviation)**	59.93 **± 8.78**
**Gender**	
Male	48 (60)
Female	32 (40)
**Comorbidity**	
Diabetes	18 (22.5)
Obesity	29 (36.35)
Cardiovascular comorbid	4 (5)
Hypertension	13 (16.25)
Stroke	4 (5)
Chronic kidney disease	4 (5)
Pulmonary Disease (chronic obstructive pulmonary disease, tuberculosis)	4 (6.25)
**Vaccination status**	
Vaccinated	11 (13.75)
Non-vaccinated	69 (86.25)

### Laboratory findings

**Figure 1.**
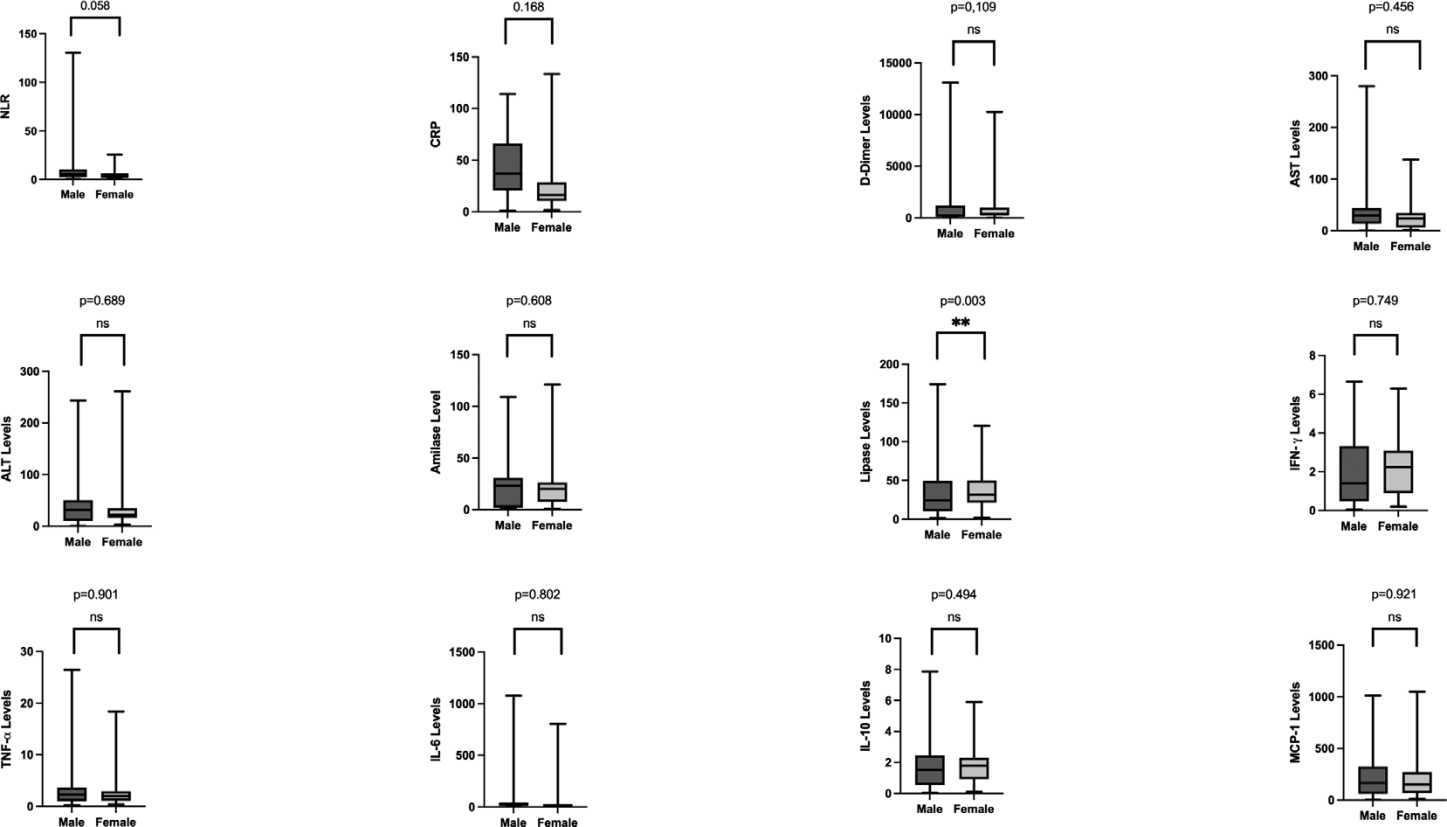
Laboratory parameters between genders. Lipase levels were significantly higher in men (129.5 (±52.32), p = 0.003). C-reactive protein, aspartate aminotransferase levels, and interferon-gamma were also higher in men. Women, though not significant, had higher levels of D-dimer, alanine Aminotransferase levels, amylase, tumour necrosis factor-alpha, interleukin-6, interleukin-10, and monocyte chemoattractant protein-1.

**Figure 2.**
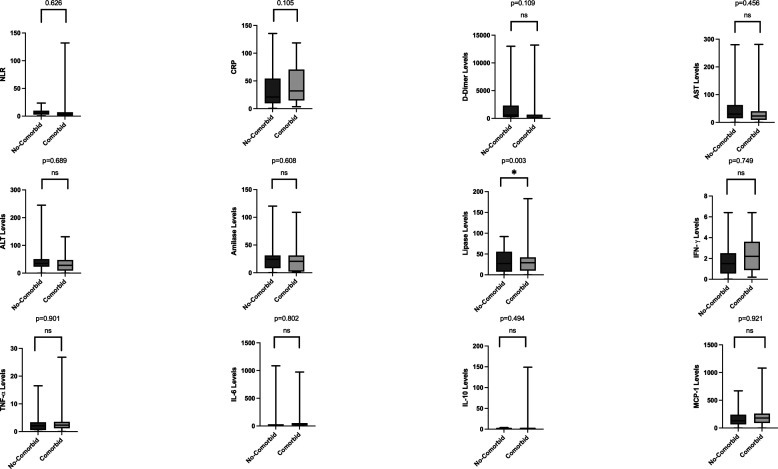
Laboratory parameters between non-comorbid and comorbid groups. Lipase levels were significantly higher in patients with comorbidities. Most laboratory parameters were higher in patients with comorbidities, except for aspartate aminotransferase levels and interferon-gamma.

**Figure 3.**
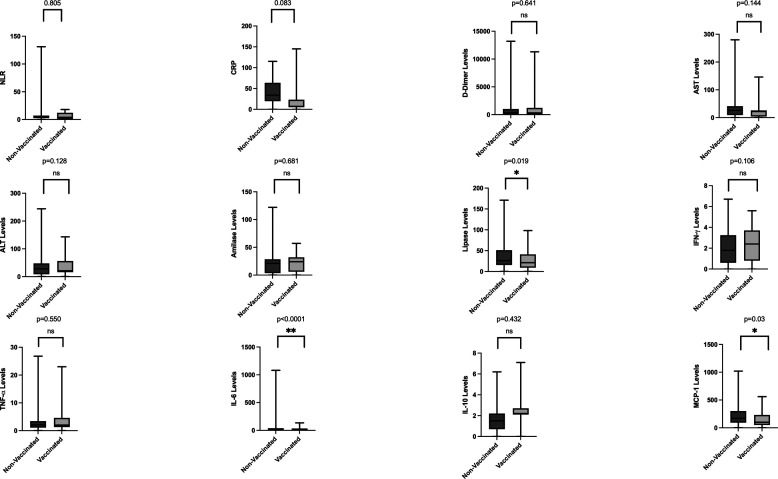
Laboratory parameters between non-vaccinated and vaccinated groups. Lipase levels, interleukin-6 levels, and monocyte chemoattractant protein-1 levels were significantly lower in vaccinated patients ( *p-*values = 0.019, <0.0001, and 0.03, respectively). Also, aspartate aminotransferase levels and amylase levels also were lower in vaccinated subjects.

Table 2 presents the laboratory results from this study. The leucocyte and neutrophil percentages increased in the subjects. The neutrophil to lymphocyte ratio (NLR) was 7. The inflammatory findings were increased in severe COVID-19 patients compared with normal value in the study. D-dimer as a coagulopathy parameter increased above the normal range in this study. The subjects also had increased CRP, ALT levels, AST levels, and lipase. Other parameters including serum electrolyte levels and renal function, were within normal reference values. Results of comparison analysis between males and females are shown in Figure 1. Lipase levels were higher in men (129.5 (±52.32), p = 0.003). Analyses of BMI groups are presented in Table 3. There are no significant differences found between different BMI groups. As for the analysis regarding non-comorbid and comorbid groups, lipase levels were higher in groups with comorbidity compared with those without comorbidity (shown in Figure 2). Between the vaccinated and non-vaccinated groups, results indicated a significantly higher level of lipase, IL-6, and MCP-1 ( *p-*values = 0.019, <0.0001, and 0.03, respectively) in the non-vaccinated group ( Figure 3).

**Table 2.** Clinical and laboratory findings of severe SARS-CoV-2.

Variables	Baseline	Normal value
BMI (Body Mass Index) (kg/m ^2^)	23.60 (23.40-24.60)	18.5-23
Hemoglobin (g/dL)	12.25 (11.80-13.00)	12.5-16.3
Leukocyte (per mm ^3^)	11,300 (9,990-12,300)	4,000-10,200
Thrombocyte (per mm ^3^)	294,000 (257,000-332,000)	150,000-450,000
Neutrophil (%)	80.70 (78.70-84.60)	55-70
Lymphocyte (%)	12.20 (8.5-15.1)	20-40
Monocyte (%)	4.6 (4.2-5.8)	2-8
C-reactive protein (mg/L)	77 (64-96)	<10
Sodium/Na (mmol/L)	138.5 (135-140)	135-145
Potassium/K (mmol/L)	4.5 (2.8-7.2)	3.5-5
Chloride (mEq/L)	105.5 (91-119)	95-105
Calcium (mmol/L	8.9 (8.8-9.2)	8.6-10.3
Urea (mg/dL)	44 (33-54)	15-40
Creatinine (mg/dL)	1.12 (0.94-1.35)	0.7-1.2
D-Dimer (ng/mL)	800 (760-980)	<500
Aspartate Aminotsransferase (U/L)	55.5 (44-65)	<31
Alanine Aminotransferase (U/L)	67 (46-73)	<32
Amylase (U/L)	57 (50-58)	19-86
Lipase (U/L)	113.5 (106-135)	7-59
Interferon-Gamma (pg/mL)	4.4 (4-5)	<4.2
Tumour necrosis factor-alpha (pg/mL)	7.3 (6.2-8.4)	<2.8
Interleukin-6 (pg/mL)	43.5 (32-57)	<7.0
Interleukin-10 (pg/mL)	5.8 (5.2-6.8)	<3.5
Monocyte chemoattractant protein-1 (pg/mL)	380 (295-455)	<300

All data presented as median (interquartile range).

**Table 3.** Inflammation laboratory findings of hospitalised patients infected with SARS- Cov-2 stratified by body mass index.

Parameter	Underweight	Norm weight	Overweight	Obese	p-value
Neutrophil-lymphocyte ratio	3.5 (1.5-10.75)	7.0 (3.0-18.0)	9.5 (4.5-15.0)	6.0 (2.0-15.5)	0.472
C-reactive protein	67.0 (59.25-133.3)	70.0 (55.0-148.0)	92.5 (64.25-151.0)	58.0 (49.5-147.0)	0.632
D-dimer	2180 (377.5-11.400)	900 (560-1800)	840 (540-2750)	800 (500-1475)	0.939
Alanine Aminotransferase	44.5 (30.75-264.5)	55 (34-79)	70 (27.3-129.0)	54 (30.5-78.0)	0.656
Aspartate Aminotransferase	34 (19.50-86.0)	67 (36-88)	62.5 (32.5-127.3)	69.0 (33.5-98.0)	0.697
Amylase	66 (42.0-88.5)	55 (34-66)	67.0 (55.2-84.7)	53.0 (33.5-98.0)	0.072
Lipase	154 (120.5-177.0)	115 (106-186)	109.5 (70.5-140.8)	106.0 (82.0-138.0)	0.246
Interferon-gamma	3.7 (2.17-9.05)	4.9 (3.2-7.8)	4.35 (3.02-5.8)	4.3 (2.35-7.10)	0.667
Tumour necrosis factor-Alpha	5.6 (5.07-19.33)	7.9 (6.0-11.9)	7.35 (5.17-10.08)	7.3 (5.05-10.0)	0.676
Interleukin-6	26.0 (20.7-854.0)	49.0 (26.0-78.0)	44.5 (25.7-71.5)	43.0 (21.5-100)	0.964
Interleukin-10	5.3 (4.5-5.4)	6.2 (4.6-7.9)	5.5 (4.0-7.75)	6 (4.3-8.6)	0.632
Monocyte chemoattractant protein-1	275.0 (222.5-650)	315 (185-505)	460 (352.5-602.5)	355.0 (185.0-580)	0.232

All data presented as median (interquartile range).

## Discussion

More men were included in our study group with severe COVID-19, suggesting that they suffer from the severe form of COVID-19 compared with women. Also, recent studies suggested that men also tend to present more severe forms of the disease and have a higher mortality rate. ^28^ The number of men who died is 2.4 times that of women. While men and women had the same susceptibility, men were more prone to dying. ^28^^,^^29^ Potential risk factors have been suggested, including different behaviours between genders, genetic and hormonal factors, and the influence of sex genetics in viral pathogenesis. ^29^ Risky behaviours such as smoking and alcohol consumption have been reported in more men than women. ^30^ These behaviours increase risks for hypertension, cardiovascular disease, and chronic pulmonary disease, which could exacerbate the severity and susceptibility to COVID-19. The mechanism of SARS-CoV-2 infection is regulated by the expression of ACE-2 and TMPRSS2 genes. These factors are often associated with sex. For instance, the ACE-2 gene is found on the X-chromosome. Inactivation of this gene has been associated with the incidence of COVID-19 in males and females. ^31^ The gene that transcribes TMPRSS2 is influenced by androgens, and the presence of androgens promotes the expression of TMPRSS2. TMPRSS2 acts as a co-receptor for SARS-CoV-2 cell invasion, and its high expression will lead to increased susceptibility for COVID-19. ^32^^,^^33^

The mean age of all the subjects in our study was 59 years old, with the youngest subject over 50 years old. Older people were more sensitive to SARS-CoV-2 infection and had a higher positive rate than younger individuals. ^34^ Also, older subjects were associated with increased mortality from COVID-19 due to poorer lung function and the likelihood of having comorbidities when compared with younger patients. ^34^^,^^35^ Ageing is associated with an increase in pro-inflammatory cytokine levels (CRP, D-dimer, procalcitonin, and IL-10). ^36^ Older patients typically experience a decline in physiological immune function and immunosuppression, thus making it difficult for them to control pro-inflammatory responses. ^35^

Obesity is one comorbidity related to severe COVID-19. ^37^ Subjects with a BMI of less than 18.5 kg/m ^2^ and greater than 25 kg/m ^2^ have a higher risk for a fatal illness. ^38^ In this study, a total of 36.35% were obese and 6.25% were underweight based on Asian BMI criteria. Obesity is one of the risk factors for cardiometabolic disease and is reported to cause immune system dysregulation. Obese patients have the highest risk for longer hospitalisation and death due to COVID-19. ^39^ Adipocytes could increase the inflammatory response by stimulating macrophages to produce interleukins (IL-1, IL-6, IL-8, IL-10) and TNF-alpha. Meanwhile, underweight COVID-19 patients are at risk of developing acute kidney injury, worsening the patient’s condition. ^40^ In this study, laboratory parameters were not significantly different within various BMI groups. This might also be influenced by other patient condition.

Diabetes mellitus is also widely associated with the incidence of COVID-19. ^41^ Increased glucose metabolism in patients with diabetes could directly enhance the replication of SARS-CoV-2. Increased glucose escalates the production of mitochondrial reactive oxygen species and activates hypoxia-inducible factor 1α. ^42^ Insulin resistance itself is associated with an impaired response to IFN type 1, thus generating a high viral load and inhibiting the body’s inflammatory response. ^43^

Vaccination is part of the prevention program against SARS-CoV-2. The vaccine promotes antibody production to prevent COVID-19. ^44^ Also, vaccine administration effectively reduces disease severity. ^23^ In this study, 86.25% of patients were reported as non-vaccinated. Several parameters were significantly different between the vaccinated and non-vaccinated groups. Lipase, IL-6, and MCP-1 were higher in the non-vaccinated group. Increased lipase and IL-6 indicate an inflammatory response and a more severe disease. ^7^ On the other hand, MCP-1 is suspected of inhibiting of IFN-signalling. ^45^ IFNα and IFN-β have antiviral activity; thus non-vaccinated individuals tend to have poorer immune response due to low antiviral activity. ^46^

Subjects in this study had an increased leukocyte count and lymphopenia. This finding was also observed in the meta-analysis by Huang *et al*., which reported that patients with severe COVID-19 tended to have higher leukocyte counts and lower lymphocyte counts compared to non-severe illness. ^47^ Leucocytosis may be present due to co-infection with bacterial pneumonia. Steroid medication given to those with severe illness induces leucocytosis or variability in the immune response. ^48^ Lymphopenia might be directly induced by lymphoid tissue destruction, inflammatory cytokines or a metabolic disorder that caused by COVID-19 infection. TNF-alpha, IL-6, and other inflammatory cytokines could induce a lymphocyte deficiency. ^49^

Neutrophilia was also reported in this study, with an NLR of 7. In patients with COVID-19, NLR may reflect the severity of inflammation. Neutrophil percentages have been mainly within the normal range in non-severe cases but were increased in the severe form of illness. ^48^ Older and critical patients tend to present with neutrophilia, suggesting that this condition related to the cytokine storm. ^50^^,^^51^ A predictive risk model by Liu *et al*. suggested that the incidence of severe disease was 50% in patients with aged 50 years or older and NLR of greater than or equal to 3.13 compared with 9.1% in patients with an age 50 years or older and NLR of less than 3.13. ^52^ In our study, subjects were >50 years with an NLR greater than 3.13, thus correlating with the risk of severe disease.

Inflammatory parameters (IL-6, IL-10, TNF-alpha, IFN-gamma, MCP-1) were increased in COVID-19 patients with severe illness. These findings were also found in recent studies. ^16^^,^^20^^,^^24^^,^^53^^,^^54^ Patients with COVID-19 had high amounts of pro-inflammatory cytokines (IFN-gamma, TNF-alpha, IP-10, IL-1B, MCP-1). Patients requiring ICU admission had higher cytokines, suggesting that a cytokine storm was associated with ARDS progression and severe illness. However, COVID-19 patients also present with increased anti-inflammatory cytokines (IL-4, IL-10), that differ from those in SARS-CoV infection. ^24^^,^^53^ The Univariate Cox Analysis by Yang *et al*. indicated that circulating IL-6 significantly predicted the progression of COVID-19 infection. Serum IL-6 was higher in COVID-19 patients with pneumonia than those without pneumonia. Increased IL-6 might induce tissue-damaging-inflammation and cause alveolar cell injury. ^20^ Patients with IL-6 levels greater than 32.1 pg/mL were more likely to have severe complications. ^16^ IL-6 trans-signalling could enhance the production of IL-8, MCP-1, and IL-10. ^54^

D-dimer also increased in our subjects, with a mean of 800 ng/mL. D-dimer is a fibrin degradation product widely used as a biomarker for thrombotic disorders. D-dimer value of less than 500 ng/mL is usually considered normal. D-dimer can predict severe and fatal cases of COVID-19 with moderate accuracy (sensitivity 77%, specificity 71%). ^55^ In a multicentre meta-analysis by Paliogiannis *et al*., D-dimer concentrations in patients with severe COVID-19 were significantly higher than those with non-severe forms. ^56^ In the analysis by Ozen *et al*., threshold D-dimer value of 370 ng/ml was calculated to have 74% specificity and 77% sensitivity for predicting lung involvement in COVID-19 patients. ^57^ A cut-off of 1500 ng/mL is the optimal value of admission D-dimer for predicting mortality in COVID-19 patients. ^58^

CRP levels increased almost eightfold above reference values in this study. CRP is an active regulator of host innate immunity and induces the classical complement pathway. Therefore, it can mediate inflammation. ^59^ A significant increase in CRP was found in COVID-19 patients, with average levels from 20 to 50 mg/L. ^60^ CRP usually is not elevated in viral infections, but the macrophage activation syndrome may explain the high serum CRP and poorer disease progression. Elevated CRP may also indicate co-infections of bacterial aetiology. ^61^ Up to 86% of patients with a severe COVID-19 had increased CRP in higher concentration than mild or non-severe patients. ^62^ The risk of developing severe events is increased by 5% for every one-unit increase in CRP levels in COVID-19 patients. ^63^

The mean level of lipase was significantly higher in men in our study. This finding was similar to the study by Barlass *et al.*, who showed that increased lipase indicated possible pancreatitis and was connected with a poor prognosis. ^64^ Although there was higher lipase activity in the male animal model, there was no definite explanation for higher lipase activity in men. ^65^ Lipase levels were also significantly different in the groups of patients with and without comorbidities. After adjusting the analysis with groups of comorbidities, there was no significant difference. Perhaps comorbidities could interfere with the body’s physiological processes and induce stress in various organs, including the pancreas.

This study has limitations because we did not compare the laboratory profiles between severe groups and mild/moderate groups. However, for COVID-19 patients, the presence of comorbidities and elevated inflammatory markers should raise healthcare providers’ awareness for the risk of severe disease course. There are also interesting results that could be important for future treatment. Lipase, IL-6, and MCP-1 results were found significantly different between the vaccine and non-vaccine groups. Elevated lipase may indicate possible pancreatic involvement that may be a consideration for managing COVID-19.

## Data availability

### Underlying data

Figshare: COVID Master Data ICU.xlsx, https://doi.org/10.6084/m9.figshare.18027170. ^66^

Data are available under the terms of the Creative Commons Zero “No rights reserved” data waiver (CC0 1.0 Public domain dedication).
