# Supplementary material for: Inflammatory laboratory findings associated with severe illness among hospitalized individuals with COVID-19 in Medan, Indonesia: a cross-sectional study
Source: F1000Res. 2022 Jan 14;10:1246. Originally published 2021 Dec 6. [Version 2] doi: 10.12688/f1000research.74758.2 (PMC8792878; doi:10.12688/f1000research.74758.2)
Supplement: Peer review history

# Peer review: Reviewer response for version 2

**DOI:** 10.5256/f1000research.119941.r119821

**Published:** 2022-01-26

**Authors:** Intan Mauli Warma Dewi

**Affiliations:** 1 Department of Internal Medicine, Radboud University Medical Center, Nijmegen, The Netherlands

Approved with no further comments.

Is the work clearly and accurately presented and does it cite the current literature?

Yes

If applicable, is the statistical analysis and its interpretation appropriate?

I cannot comment. A qualified statistician is required.

Are all the source data underlying the results available to ensure full reproducibility?

Partly

Is the study design appropriate and is the work technically sound?

Yes

Are the conclusions drawn adequately supported by the results?

Yes

Are sufficient details of methods and analysis provided to allow replication by others?

Partly

Reviewer Expertise:

NA

I confirm that I have read this submission and believe that I have an appropriate level of expertise to confirm that it is of an acceptable scientific standard.

# Peer review: Reviewer response for version 1

**DOI:** 10.5256/f1000research.78544.r102162

**Published:** 2022-01-04

**Authors:** Intan Mauli Warma Dewi

**Affiliations:** 1 Department of Internal Medicine, Radboud University Medical Center, Nijmegen, The Netherlands

With interest, I read the study entitled “Inflammatory laboratory findings associated with severe illness among hospitalized individuals with COVID-19 in Medan, Indonesia: a cross-sectional study” by Darmadi et.al. In this study, the authors present the clinical and inflammatory characteristics of critically ill COVID-19 patients in the ICU. The results highlight the increased inflammatory profiles in severe COVID-19 patients and in those with comorbidities. Please find some minor comments below:

1. In the method section, the authors mentioned that among data collected was “vaccination status”. Please elaborate whether this refers to COVID-19 vaccination (or others).
2. In the results, clinical and laboratory parameters were compared to normal reference values. Do these values represent the population where the study was performed (i.e. Indonesian?) In this case, ideally, samples measured from healthy controls and/or patients with milder disease should be included in the study to provide a better comparison.
3. In table 3, the authors compared inflammatory marker levels in patients with different BMI categories and showed no significant differences between the groups. Did the author also look at the correlation between the BMI values and levels of these markers to further justify this finding?
4. Figure 2 shows the comparison of laboratory parameters between the non-comorbid and comorbid groups. However, in this study, the assessed comorbidities have completely different mechanisms, and hence, characteristics (for example, those with pulmonary diseases such as COPD or tuberculosis, those with CKD, and those with metabolic or cardiovascular diseases would have different immune background). Can the authors justify why in figure 2 the comorbidities were categorized into one entity and not analyzed as separate?
5. In figure 3, is the duration post-vaccination (I would assume COVID-19 vaccination?) and numbers of vaccines known? Please elaborate in the methods section.
6. In the methods section, the authors only mentioned that samples were collected after 24 hours of ICU admission. Is the duration of illness known? The authors should add this in the methods section, or elaborate in the discussion.

Is the work clearly and accurately presented and does it cite the current literature?

Yes

If applicable, is the statistical analysis and its interpretation appropriate?

I cannot comment. A qualified statistician is required.

Are all the source data underlying the results available to ensure full reproducibility?

Partly

Is the study design appropriate and is the work technically sound?

Yes

Are the conclusions drawn adequately supported by the results?

Yes

Are sufficient details of methods and analysis provided to allow replication by others?

Partly

Reviewer Expertise:

Microbiology and immunology

I confirm that I have read this submission and believe that I have an appropriate level of expertise to confirm that it is of an acceptable scientific standard, however I have significant reservations, as outlined above.

# Response

**Published:** 2022-01-07

**Authors:** Cennikon Pakpahan

**Affiliations:** Airlangga University, Indonesia

1. Thank you for the clarification. Vaccination status refers to COVID-19 vaccination; we have added this in the latest revision.

 2. We apologize for providing ambiguous information. The normal reference values ​​in the table are the normal reference values ​​used by the laboratory during the examination.

 3. Thank you for providing interesting input on our article. We've determined the statistical tests (disease type and lab parameters) and it was an insignificant correlation with all inflammation parameters (p>0.05). We include the statistical results in the supplementary data. We just presented statistically significant data to address our study.

 4. We thank you for your opinion. At the beginning of the study, we conducted this test. However, we found no significant results between the comorbid and inflammatory parameters. Then we tried to group all types of comorbidity into one group and compared with no comorbidity. From this test, several significant parameters were obtained. We include the statistical results of this test in the supplementary data.

 5. We recognize that this information is essential. However, many patients who enter our hospital are in poor condition, so we do not get factual information about this. We decided not to include this in the study to prevent recall bias.

 6. Once again, thank you very much for your opinion. The problem we found with this information was that some of our research samples were in poor condition, not worthy of being asked for information. To prevent recall bias, we decided not to include it.

# Response

**Published:** 2022-01-19

**Authors:** Intan Dewi

**Affiliations:** Radboud University Nijmegen Medical Centre, The Netherlands

The authors have sufficiently addressed the comments. I have no further comments on the revised version of the manuscript.

# Peer review: Reviewer response for version 1

**DOI:** 10.5256/f1000research.78544.r102157

**Published:** 2021-12-21

**Authors:** Kurnia Fitri Jamil

**Affiliations:** 1 Department of Orthopaedics and Traumatology, Universiti Kebangsaan Malaysia, Kuala Lumpur, Indonesia

The suggestions that we can give to the manuscript are:

1. The grammatical errors in the text were quite large in number. Authors need to send the manuscript for proofreading and correction.
2. As highlighted by the authors, the time of study was short.
3. It is better if the authors can provide more updates or additional facts that can be obtained from this study compared to what has already been published in the literature.
4. I suggest that the inflammation laboratory findings, especially C-reactive protein, are compared when the patient is admitted to the hospital and at the end of the treatment, and differentiated by each stage of COVID-19 disease.
5. References: Correct or full citation for reference numbers 2, 15, 20, 36, 50, 54, 62.

Is the work clearly and accurately presented and does it cite the current literature?

Yes

If applicable, is the statistical analysis and its interpretation appropriate?

Yes

Are all the source data underlying the results available to ensure full reproducibility?

Yes

Is the study design appropriate and is the work technically sound?

Yes

Are the conclusions drawn adequately supported by the results?

Yes

Are sufficient details of methods and analysis provided to allow replication by others?

Partly

Reviewer Expertise:

Tropical disease and infection, Internal medicine

I confirm that I have read this submission and believe that I have an appropriate level of expertise to confirm that it is of an acceptable scientific standard.

# Response

**Published:** 2022-01-01

**Authors:** Cennikon Pakpahan

**Affiliations:** Airlangga University, Indonesia

1. In response to the reviewer’s comments, we have sent and revised the manuscript according to Enago English Editing and Proofreading Services.

 2. Thank You for your valuable comment. We conducted a cross-sectional study to explore the inflammatory profile of severe COVID-10 patients. Hence, we only involved looking at data from COVID-19 cases admitted to the ICU of Mitra Medica General Hospital Medan at one specific point in time (between May and June 2021).

 3. We thank your reviewer’s suggestion. During our research and writing the manuscript, there are not many studies had reported on inflammatory parameters, enzymes, and COVID-19 prognosis. We cited new findings, such as the inflammatory profile of severe COVID-19 patients with or without vaccination. We think these things are new facts regarding COVID-19 management.

 4. Thank you for the interesting comment. We apologize for not presenting these data, because, in the beginning, we did not design the study to compare the inflammatory profile between the degrees of COVID-19. We only aimed to explore the profile of inflammation parameters in the severe group with various variables. We have described this in the limitation of the study in the last paragraph.

 5. Once again thank you for your comment. We have rechecked for references consistency carefully.
